# Physical activity and risk of prostate and bladder cancer in China: The South and East China case-control study on prostate and bladder cancer

**DOI:** 10.1371/journal.pone.0178613

**Published:** 2017-06-02

**Authors:** Raoul C. Reulen, Stefan de Vogel, Weide Zhong, Zhaohui Zhong, Li-Ping Xie, Zhiquan Hu, Yilan Deng, Kai Yang, Yuxiang Liang, Xing Zeng, Yong Chuan Wong, Po-Chor Tam, Marjolein Hemelt, Maurice P. Zeegers

**Affiliations:** 1 Insititute of Applied Health Research, University of Birmingham, Edgbaston, Birmingham, United Kingdom; 2 Department of Public Health and Primary Health Care, University of Bergen, Bergen, Norway; 3 Department of Urology, The 1st Municipal Hospital of Guangzhou, Guangzhou Medical College, Guangzhou, China; 4 Department of Urology, The Second Xiangya Hospital, Central South University, Changsha, China; 5 Department of Urology, First Affiliated Hospital, Zhejiang University School of Medicine, Hangzhou, China; 6 Department of Urology, Tongji Hospital, Huazhong University of Science and Technology, Wuhan, China; 7 Department of Anatomy, Faculty of Medicine, The University of Hong Kong, Hong Kong, China; 8 Department of Surgery, The University of Hong Kong, Hong Kong, China; 9 Department of Complex Genetics and Epidemiology, Nutrition and Toxicology Research Institute, Maastricht University, Maastricht, The Netherlands; University of Oxford, UNITED KINGDOM

## Abstract

**Background:**

Recent meta-analyses have suggested a modest protective effect of high levels of physical activity on developing both prostate and bladder cancer, but significant heterogeneity between studies included in these meta-analyses existed. To our knowledge, few Chinese studies investigated the association between physical activity and prostate cancer and none between physical activity and bladder cancer. Given the inconsistencies between previous studies and because studies on the relation between physical activity and prostate and bladder cancer in China are scarce, it remains elusive whether there is a relationship between physical activity and prostate and bladder cancer within the Chinese population.

**Methods:**

We investigated the association between physical activity and risk of developing prostate and bladder cancer within a hospital-based case-control study in the East and South of China among 260 and 438 incident prostate and bladder cancer cases, respectively, and 427 controls. A questionnaire was administered to measure physical activity as metabolic equivalents (METs). Random effects logistic regression was used to calculate odds ratios (ORs) of prostate and bladder cancer for different levels of physical activity and for the specific activities of walking and cycling.

**Results:**

Increasing overall physical activity was associated with a significant reduction in prostate cancer risk (P_trend_ = 0.04) with the highest activity tertile level showing a nearly 50% reduction in prostate cancer risk (OR = 0.53, 95%CI: 0.28–0.98). Overall physical activity was not significantly associated with risk of bladder cancer (P_trend_ = 0.61), neither were vigorous (P_trend_ = 0.60) or moderate levels of physical activity (P_trend_ = 0.21). Walking and cycling were not significantly associated with either prostate (P_trend_> = 0.62) or bladder cancer risk (P_trend_> = 0.25).

**Conclusions:**

The findings of this largest ever case-control study in China investigating the relationship between physical activity and prostate and bladder cancer suggest that overall physical activity is associated with a decreased risk of prostate cancer, but not with bladder cancer.

## Introduction

The incidence of prostate and bladder cancer varies widely across the world. Highest incidence rates for prostate and bladder cancer are generally observed in Western countries (~70 and 17 cases per 100,000 per year, respectively) and the lowest incidence rates are generally observed in Eastern Asia including China (~11 and 6 cases per 100,000 per year, respectively) [1]. Potential reasons for this variation between countries in incidence rates may relate to differences in cancer registration practices, diagnostic practices (e.g. prostate specific antigen testing for prostate cancer), environment, genetic factors or lifestyle factors such as diet and physical activity [2]. Physical activity is a modifiable lifestyle factor that may reduce cancer risk, although biological mechanisms through which it would influence carcinogenesis are not fully understood [3].

Previous meta-analyses on commonly diagnosed cancers found inverse associations between physical activity and colon cancer [4–6], lung cancer [7, 8], and breast cancer [9, 10]. Liu *et al* [11] systematically reviewed studies investigating the association with prostate cancer, which were predominantly conducted in the USA, Canada, and Europe. The summary relative risk (RR) of prostate cancer was 0.90 (95% confidence interval (CI): 0.84–0.95) when comparing the most active to the most sedentary men across studies, but there was significant heterogeneity between studies. Thus far, to our knowledge, only four case-control studies investigated the relationship between physical activity and prostate cancer in a Chinese population with varying results; two studies reported an inverse association with physical activity, one an increased association and one a null-association [12–15].

With regard to bladder cancer, a recent meta-analysis on the association of physical activity and bladder cancer identified a 15% decreased risk (RR = 0.85, 95%CI: 0.74–0.98) of developing bladder cancer among individuals with high levels of physical activity compared to low activity, but there was considerable heterogeneity between the 15 studies included in this meta-analysis [16]. To our knowledge, no study investigated the association between physical activity and bladder cancer in China.

Given the inconsistencies between previous studies and because studies on the relation between physical activity and prostate and bladder cancer in China are scarce, it remains elusive whether there is a relationship between physical activity and prostate and bladder cancer within the Chinese population. Because the incidence of prostate and bladder cancer is much lower in China than in Western countries and levels of physical activity differ as well—e.g. the adult urban Chinese population engages much less in leisure time physical activity than Western populations [17–19]—results from Western studies may not necessarily be translatable to the Chinese population.

Therefore, we conducted a hospital-based case-control study in the South and East of China to investigate whether physical activity is associated with prostate and bladder cancer. The study included 260 prostate cancer and 438 bladder cancer cases—which are considerably large numbers given the low prostate and bladder cancer incidence in China—thereby representing the largest case-control study on the association between prostate and bladder cancer and physical activity in China conducted to date.

## Methods

### Case and control ascertainment

A hospital-based case-control study was conducted between October 2005 and April 2009 in four hospitals in different provinces in the South and East of China. These hospitals were: The First Affiliated Hospital in Hangzhou, the First Municipal Hospital in Guangzhou, the Tongji Hospital in Wuhan, and the Second Xiangya Hospital in Changsha. Newly diagnosed, incident prostate (ICD-10: C61) and bladder (ICD-10: C67) cancer cases admitted to one of these hospitals were eligible for inclusion in the current study. To minimize potential selection bias due to controls having diseases that might have shared risk factors with prostate and/or bladder cancer, controls were recruited among patients who were admitted to different wards of the same hospitals within the recruitment period. Controls who had been admitted to hospital for urologic conditions, neoplastic related diseases, smoking-related diseases or Alzheimer’s disease were not eligible for inclusion. Females were eligible to be selected as a control, but were only used in analyses relating to bladder cancer. The study was approved by the relevant ethical committee of The First Affiliated Hospital, the ethical committee of the First Municipal Hospital, the ethical committee of the Tongji Hospital, the ethical committee of the Second Xiangya Hospital, and by the *National Medical Information Profession Committee* of China. Participants received information about the objectives of the study and provided written informed consent prior to enrollment.

### Measurement of physical activity

Trained interviewers administered a computerised questionnaire to obtain information about physical activity according to the short form of the International Physical Activity Questionnaire (IPAQ) [20] (see also: www.ipaq.ki.se). The questions related to all physical activity during an average week, one year prior to the interview. Information was obtained on type (*e*.*g*. vigorous and moderate physical activity, walking and cycling) and duration of physical activity across domains spanning occupational and non-occupational physical activity. Age at questionnaire completion, height, weight, smoking history, highest educational level achieved, and information on health insurance were obtained through the same questionnaire.

### Statistical analysis

Data processing and analyses of physical activity questions were performed following the IPAQ guidelines. Physical activity was calculated as Metabolic Equivalent hours per week (MET-h/wk) by weighting each type of activity by its energy requirements. To facilitate comparison of MET-h/wk to previous Chinese studies on physical activity and prostate cancer a similar weighting was used in which the weights 5 and 3 were used for vigorous activity and moderate activity, respectively [12, 13]. The standard weighting scores of 8 and 4 generally used are based on a young adult population and are therefore likely to be too high for an elderly study population [21]. METs were subsequently categorized as low, medium, and high physical activity based on the nearest tertile cut-off values in the distribution among controls. A random-effects unconditional logistic regression model was used to estimate odds ratios (ORs) of developing prostate and bladder cancer for different levels of physical activity. To account for potential dependencies between participants recruited from the same hospital, the factor hospital was incorporated as a random effect in the model. Models were adjusted for the covariates: age at interview completion (continuous), sex (for bladder cancer only), height (continuous), weight (continuous), smoking status (never, ex, and current smokers), number of cigarettes smoked per day (continuous), highest achieved educational level (none/primary school/junior school/technical/senior technical/university), and whether individuals had health insurance (yes/no). For the physical activity variables and continuous covariates departures from linearity were evaluated using restricted cubic splines [22]. No significant non-linearity was detected for any of the covariates, apart from age at interview completion which was fitted in the logistic regression model using a cubic spline with six knots.

Sensitivity analyses were conducted by stratifying the results by age at diagnosis (<65 years *vs*. > = 65 years). A test for interaction was performed to assess whether the ORs for the different age groups differed significantly between these strata. All analyses were performed using Stata 14 (StataCorp, College Station, Texas). A two-sided P-value < .05 was considered statistically significant.

## Results

### Case and control characteristics

The study included 260 prostate, 438 bladder cancer patients and 427 controls with response rates of 94%, 94% and 96%, respectively. Prostate cancer cases (mean = 72.0 yrs) were generally older than controls (mean = 65.1 yrs), but bladder cancer cases (mean = 63.6 yrs) were of similar age as controls (Table 1). Mean BMI was similar for both prostate (mean = 23.1) and bladder (mean = 23.0) cancer cases compared to controls (mean = 23.2). Bladder cancer cases were more likely to be current smokers (41.3%) than controls (26.2%) or prostate cancer cases (25.0%).

**Table 1:** Characteristics of prostate and bladder cancer cases and controls in the South and East China case control study on prostate and bladder cancer (SEARCH).

		Prostate cancer cases	Bladder cancer cases	Controls
**overall**		260 (100.0%)	438 (100.0%)	427 (100.0%)
**sex**	male	260 (100.0%)	352 (80.4%)	336 (78.7%)
	female	0 (0.0%)	86 (19.6%)	91 (21.3%)
**age (yrs)**	Mean (SD)	72.0 (8.1)	63.6 (13.8)	65.1 (13.1)
	<60	20 (7.7%)	174 (39.7%)	143 (33.5%)
	60–69	73 (28.1%)	91 (20.8%)	109 (25.5%)
	70–79	119 (45.8%)	128 (29.2%)	132 (30.9%)
	80+	48 (18.5%)	45 (10.3%)	43 (10.1%)
**BMI (kg/m**^**2**^**)**	Mean (SD)	23.1 (2.8)	23.0 (3.5)	23.2 (3.2)
	<20	28 (10.8%)	65 (14.8%)	47 (11.0%)
	20–24	180 (69.2%)	289 (66.0%)	279 (65.3%)
	25+	49 (18.8%)	84 (19.2%)	98 (23.0%)
	missing	3 (1.2%)	0 (0.0%)	3 (0.7%)
**Smoking status**	never smoked	88 (33.8%)	154 (35.2%)	198 (46.4%)
	ex-smoker	107 (41.2%)	103 (23.5%)	117 (27.4%)
	currrent smoker	65 (25.0%)	181 (41.3%)	112 (26.2%)
**Highest**	None	15 (5.8%)	20 (4.6%)	37 (8.7%)
**Education**	Primary school	68 (26.2%)	147 (33.6%)	134 (31.4%)
	Junior chool	62 (23.8%)	104 (23.7%)	114 (26.7%)
	Technical	57 (21.9%)	94 (21.5%)	77 (18.0%)
	Senior technical	21 (8.1%)	33 (7.5%)	35 (8.2%)
	University	35 (13.5%)	38 (8.7%)	30 (7.0%)
**Health**	No	33 (12.7%)	112 (25.6%)	124 (29.0%)
**Insurance**	Yes	223 (85.8%)	315 (71.9%)	298 (69.8%)
	Missing	4 (1.5%)	11 (2.5%)	5 (1.2%)

### Physical activity–prostate cancer

**Fig 1:**
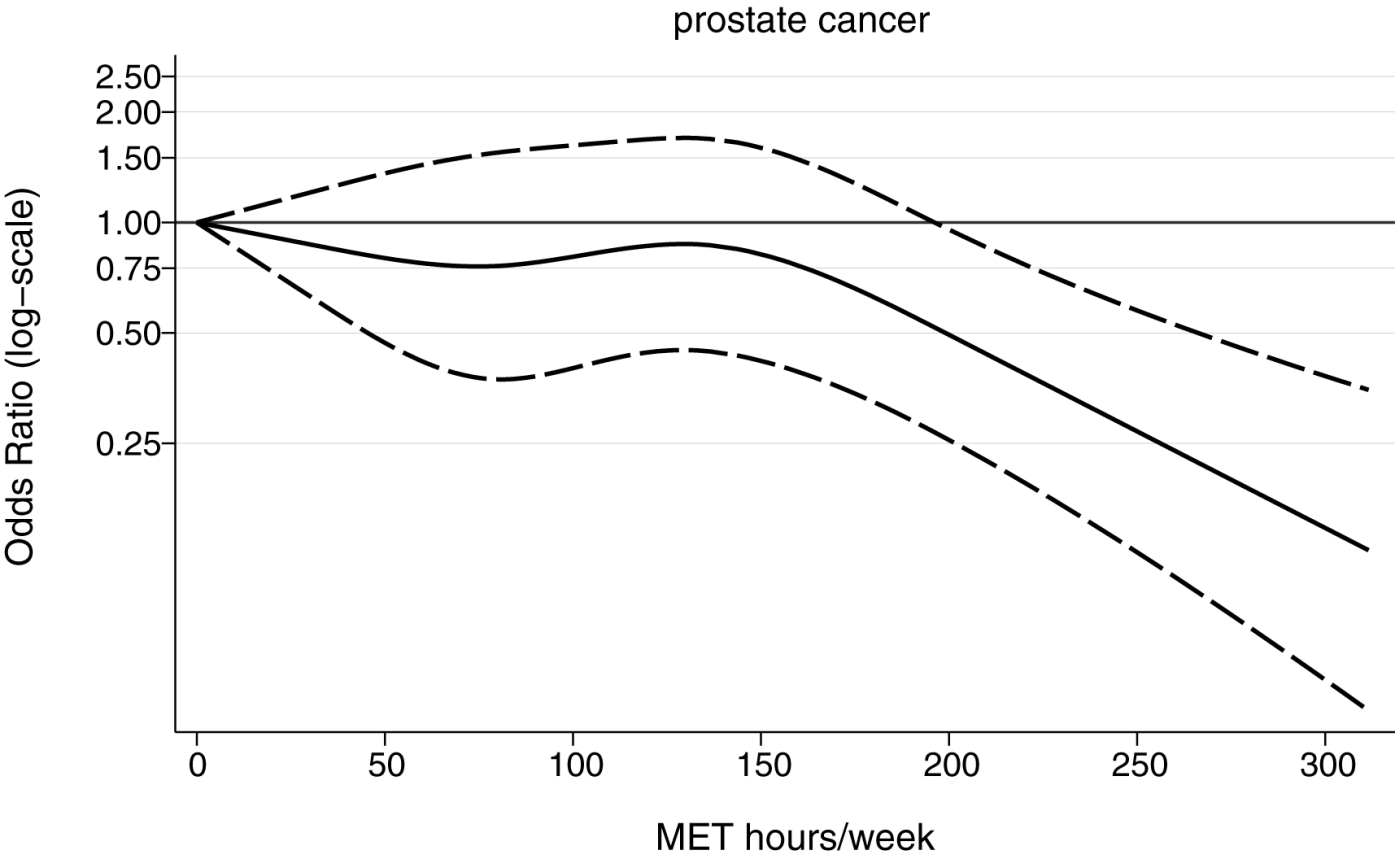
Dose-response relationship (solid line), with corresponding 95% confidence intervals (dashed lines), between overall physical activity (MET hours/Week) and odds of developing prostate cancer. Dose reponse was fitted using a restricted cubic spline with knots located at the quartiles of the MET hours/week distribution.

Increasing overall physical activity was associated with a significant reduction in prostate cancer risk (P_trend_ = 0.04) with the highest activity tertile level showing a nearly 50% reduction in prostate cancer risk (OR = 0.53, 95%CI: 0.28–0.98) (Table 2). The shape of the dose-response indicated that the OR was particularly reduced at levels of physical activity exceeding 200 MET-hours/week (Fig 1).

**Table 2:** Associations between types and intensity of physical activity and prostate cancer risk in the South and East China case control study on prostate and bladder cancer (SEARCH).

						Overall Associations		Age category (years)		
Activity^a^	Level	MET	Cases	Controls	Univariate	Age adjusted	multivariable^b^	< 65^b^	≥ 65^b^	
		(h/wk)	(N = 260)	(N = 336)	OR (95%CI)	OR (95%CI)	OR (95%CI)	OR (95%CI)	OR (95%CI)	P_het_
**All types of**	Low	< 73.5	112	111	1.00 (ref.)	1.00 (ref.)	1.00 (ref.)	1.00 (ref.)	1.00 (ref.)	
**physical**	Medium	73.5–166.2	98	113	0.70 (0.43–1.14)	0.71 (0.43–1.17)	0.69 (0.41–1.16)	0.31 (0.09–1.08)	0.80 (0.43–1.47)	
**activity**	High	166.3–383.3	50	112	0.35 (0.20–0.61)	0.54 (0.30–0.99)	0.53 (0.28–0.98)	0.58 (0.17–2.01)	0.50 (0.24–1.03)	
	*P*_trend for linearity_				<0.001	0.05	0.04	0.13	0.05	0.22
**Vigorous**	None	0	103	110	1.00 (ref.)	1.00 (ref.)	1.00 (ref.)	1.00 (ref.)	1.00 (ref.)	
**physical**	Medium	1–70	105	115	0.85 (0.54–1.32)	0.94 (0.59–1.51)	0.89 (0.55–1.45)	0.91 (0.32–2.57)	1.01 (0.57–1.77)	
**activity**	High	71–315	52	111	0.44 (0.26–0.72)	0.78 (0.44–1.38)	0.71 (0.39–1.28)	0.57 (0.20–1.61)	0.78 (0.38–1.57)	
	*P*_trend for linearity_				<0.001	0.46	0.31	0.36	0.59	0.77
**Moderate**	Low	< 42	108	93	1.00 (ref.)	1.00 (ref.)	1.00 (ref.)	1.00 (ref.)	1.00 (ref.)	
**physical**	Medium	42–62	53	90	0.46 (0.28–0.77)	0.38 (0.22–0.66)	0.30 (0.17–0.54)	0.04 (0.01–0.37)	0.39 (0.21–0.69)	
**activity**	High	63–210	99	153	0.49 (0.29–0.81)	0.52 (0.30–0.90)	0.45 (0.26–0.79)	0.38 (0.16–0.90)	0.48 (0.28–0.81)	
	*P*_trend for linearity_				0.007	0.03	0.02	0.01	0.01	0.17
**Walking**	Low	<10.5	85	57	1.00 (ref.)	1.00 (ref.)	1.00 (ref.)	1.00 (ref.)	1.00 (ref.)	
	Medium	10.5–20	48	54	1.80 (1.06–3.05)	1.54 (0.87–2.74)	1.52 (0.84–2.74)	1.04 (0.36–2.99)	1.81 (0.86–3.81)	
	High	21–84	203	149	1.30 (0.79–2.16)	1.05 (0.60–1.82)	0.93 (0.52–1.66)	0.61 (0.21–1.80)	0.87 (0.45–1.71)	
	*P*_trend for linearity_				0.44	0.93	0.62	0.37	0.39	0.63
**Cycling**	Never	—	206	326	1.00 (ref.)	1.00 (ref.)	1.00 (ref.)	1.00 (ref.)	1.00 (ref.)	
	<1 hour/wk	—	32	37	0.78 (0.40–1.52)	1.19 (0.59–2.42)	1.28 (0.61–2.67)	3.02 (1.01–9.05)	1.27 (0.50–3.19)	
	> = 1 hour /wk	—	22	64	0.42 (0.24–0.72)	1.04 (0.54–1.98)	1.18 (0.61–2.30)	0.68 (0.23–1.97)	1.56 (0.62–3.90)	
	*P*_trend for linearity_				0.002	0.93	0.65	0.41	0.35	0.38

^a^ Activity measured by International Physical Activity Questionnaire (IPAQ) short form. Categories Low, Medium, and High are based on nearest tertile cut-off points among controls.

^b^ Random effects logistic regression with hospital considered as random effect, adjusted for age, height, weight, smoking status, smoking duration and educational level, and health insurance.

Vigorous physical activity was not significantly associated with prostate cancer risk (P_trend_ = 0.31), but there was a statistically significant trend with increasing moderate physical activity and prostate cancer risk (P_trend_ = 0.02). Walking (P_trend_ = 0.62) or cycling (P_trend_ = 0.65) were not significantly associated with prostate cancer risk after adjustment for potential confounders. Stratification by age at prostate cancer diagnosis (<65 vs. > = 65 years) did not reveal any significant heterogeneity in the ORs (all P_heterogeneity_ > = 0.17).

### Physical activity–bladder cancer

Overall, physical activity was not significantly associated with risk of bladder cancer (P_trend_ = 0.61), neither were vigorous (P_trend_ = 0.60) or moderate levels of physical activity (P_trend_ = 0.21) (Table 3). Specific activities of walking (P_trend_ = 0.53) or cycling (P_trend_ = 0.25) were not significantly associated with bladder cancer risk either. ORs for all physical activity variables did not seem to vary significantly by age at bladder cancer diagnosis (all P_heterogeneity_ > = 0.14).

**Table 3:** Associations between types and intensity of physical activity and bladder cancer risk in the South and East China case control study on prostate and bladder cancer (SEARCH).

						Overall Associations		Age category (years)		
Activity^a^	Level	MET	Cases	Controls	Univariate	Age & sex adjusted	multivariable^b^	< 65^b^	≥ 65^b^	
		(h/wk)	(N = 438)	(N = 427)	OR (95%CI)	OR (95%CI)	OR (95%CI)	OR (95%CI)	OR (95%CI)	P_het_
**All types of**	Low	< 63	153	132	1.00 (ref.)	1.00 (ref.)	1.00 (ref.)	1.00 (ref.)	1.00 (ref.)	
**physical activity**	Medium	63–164.4	124	147	0.83 (0.57–1.21)	0.83 (0.57–1.22)	0.80 (0.54–1.20)	0.73 (0.41–1.31)	0.99 (0.54–1.83)	
	High	164.5–416.5	161	148	1.20 (0.77–1.88)	1.14 (0.71–1.81)	1.08 (0.65–1.80)	0.77 (0.40–1.46)	1.38 (0.65–2.92)	
	*P*_trend for linearity_				0.23	0.45	0.61	0.38	0.26	0.95
**Vigorous**	None	0	151	162	1.00 (ref.)	1.00 (ref.)	1.00 (ref.)	1.00 (ref.)	1.00 (ref.)	
**physical activity**	Medium	0.8–70	151	133	1.42 (0.98–2.07)	1.39 (0.95–2.03)	1.39 (0.94–2.07)	1.34 (0.74–2.42)	1.96 (1.07–3.59)	
	High	71–315	136	132	1.46 (0.96–2.22)	1.32 (0.83–2.08)	1.23 (0.75–2.02)	0.70 (0.38–1.29)	2.17 (1.02–4.60)	
	*P*_trend for linearity_				0.15	0.35	0.60	0.20	0.11	0.80
**Moderate**	Low	< 42	160	123	1.00 (ref.)	1.00 (ref.)	1.00 (ref.)	1.00 (ref.)	1.00 (ref.)	
**physical activity**	Medium	42–62	90	102	0.78 (0.51–1.19)	0.78 (0.51–1.20)	0.73 (0.46–1.15)	0.84 (0.45–1.58)	0.63 (0.30–1.31)	
	High	63–210	188	202	0.80 (0.53–1.21)	0.77 (0.51–1.17)	0.74 (0.48–1.16)	0.65 (0.37–1.15)	0.71 (0.34–1.51)	
	*P*_trend for linearity_				0.31	0.23	0.21	0.14	0.52	0.54
**Walking**	Low	<10.5	124	108	1.00 (ref.)	1.00 (ref.)	1.00 (ref.)	1.00 (ref.)	1.00 (ref.)	
	Medium	10.5–20	69	68	0.87 (0.56–1.34)	0.91 (0.58–1.41)	0.83 (0.52–1.32)	0.75 (0.40–1.41)	0.92 (0.46–1.85)	
	High	21–73.5	245	251	0.95 (0.63–1.43)	0.99 (0.65–1.49)	0.86 (0.55–1.32)	0.92 (0.51–1.64)	0.72 (0.38–1.37)	
	*P*_trend for linearity_				0.86	0.99	0.53	0.84	0.30	0.26
**Cycling**	Never	—	312	326	1.00 (ref.)	1.00 (ref.)	1.00 (ref.)	1.00 (ref.)	1.00 (ref.)	
	<1 hour/wk	—	48	37	1.34 (0.81–2.20)	1.35 (0.82–2.24)	1.23 (0.72–2.09)	0.79 (0.39–1.59)	1.98 (0.85–4.62)	
	> = 1 hour /wk	—	78	64	1.42 (0.97–2.08)	1.41 (0.90–2.19)	1.32 (0.82–2.13)	1.05 (0.58–1.90)	1.10 (0.40–3.03)	
	*P*_trend for linearity_				0.07	0.13	0.25	0.82	0.72	0.14

^a^ Random effects logistic regression with hospital considered as random effect, adjusted for age, sex, height, weight, smoking status, smoking duration, educational level, and health insurance

^b^ Activity measured by International Physical Activity Questionnaire (IPAQ) short form. Categories Low, Medium, and High are based on nearest tertile cut-off points among controls.

## Discussion

### Principal findings

In this largest ever case-control study in China investigating the relationship between physical activity and prostate and bladder cancer, we found that overall physical activity, and in particular moderate levels of activity, is associated with a decreased risk of prostate cancer, but bladder cancer is not.

### Previous studies

Given that studies typically have used different domains of physical activity such as total physical activity, occupational, or non-occupational physical activity, it is difficult to directly compare our findings with previous studies on physical activity and prostate and/or bladder cancer. In this study, activity levels among male controls (mean = 128.7 ± 91.4 MET-h/wk) were higher, but not inconsistent to that observed in individuals of a similar age of the general population of China (mean = 109.2 ± 93.1 MET-h/wk) [17]. Our results in relation to prostate cancer are consistent with the reduced risk observed with increasing physical activity and prostate cancer from a recent meta-analysis [11]. Similar to our study, two previous Chinese studies reported total physical activity in METs using short forms of the IPAQ [12, 13]. One of those studies did not show an association with prostate cancer [13], but activity levels observed were typically higher than in our study which might have obscured any effect of physical activity on prostate cancer, i.e. there might have been a lack of contrast between the different physical activity exposure categories. However, in the one study [12] that did show a protective effect of physical activity cases and controls had similar levels of observed physical activity as in our study.

Our results of a null-association between physical activity and bladder cancer are not inconsistent with the summary relative risk of 0.85 (95%CI: 0.74–0.98) reported in a recent meta-analysis on physical activity and bladder cancer since the confidence intervals of the ORs reported in our study overlap with those reported in this meta-analysis. Although to our knowledge no other study on physical activity and bladder cancer has been conducted in China, one study conducted in an Asian population (South-Korea) also did not find any significant association [23]. We acknowledge that the evidence presented in this single study remains insufficient to conclude that there is definitely no association between physical activity and bladder cancer in a Chinese population; however, given the size of our study population, if there is an undetected association between physical activity and bladder cancer it is likely to be small.

### Biological mechanism

Although the underlying mechanism for the reduced risk of prostate cancer in physically active men remains elusive, suggestions have included a reduction in testosterone levels, prevention of obesity, enhanced immune system and reduction of oxidative stress [24, 25]. However, there have been to-date no studies that have demonstrated conclusive evidence supporting any of these postulated mechanisms. Alternatively, studies might not have been able to fully adjust for unmeasured or difficult to measure confounders, such as lifestyle factors, which might have given rise to the apparent protective association between physical activity and prostate cancer [24]. Active men probably have a generally healthier lifestyle than inactive men and therefore potential reduced risk of developing cancer; although the evidence that lifestyle, including diet and smoking, is associated with prostate cancer is limited.

### Study strengths and limitations

It has been suggested that physically active men are more likely to undergo PSA testing [11], particularly in Western developed countries. Since most prostate cancers are asymptomatic, PSA testing will typically detect prostate cancers that otherwise would have gone undetected for many years. If true, this form of diagnostic bias might explain why some studies in Western developed countries did not find an association between physical activity and prostate cancer. One advantage of our study is that PSA testing is uncommon in China avoiding this potential diagnostic bias.

A potential limitation of any case-control study is the occurrence of reverse causation which may result in under- or overestimation of associations if exposure levels are not independent from the disease outcome. Although a prospective cohort setting would not have such a methodological disadvantage, this would require a very large cohort size and long follow-up period given the low incidence rate of prostate and bladder cancer in China [1]. In this study, participants were asked to report habitual physical activity one year prior to the diagnosis of prostate cancer. This approach should have minimized reverse causation, although no information is available from literature as to whether pre-clinical prostate cancer affects physical activity prior to its clinical manifestation.

Lastly, despite the use of the internationally acknowledged and validated IPAQ short form questionnaire and use of trained interviewers, the measurement of physical activity we used may still have been affected by misclassification. The IPAQ short form has been internationally validated in over 20 studies worldwide [26]. In a large number of these validation studies the IPAQ short form overestimated physical activity when compared to more objective measures such as the accelerometer [26]—although a study specifically conducted among elderly Chinese showed in fact good validity [27]. Although the lack of evidence supporting the validity of the IPAQ short form is of concern, even if misclassification in terms of an overestimation of physical activity would be present in our study there are no strong grounds for assuming that misclassification would be more common among cases than controls or *vice versa*. Any misclassification would most likely be non-differential and thus any relative measures—such as the odds ratio—we report should largely have been unaffected, however, absolute measures of physical activity should be interpreted cautiously.

### Conclusion

In conclusion, the findings of this largest ever case-control study in China investigating the relationship between physical activity and prostate and bladder cancer suggest that overall physical activity is associated with a decreased risk of prostate cancer, but not with bladder cancer.
